# Targeted treatment of T-cell acute lymphoblastic leukemia: latest updates from the 2022 ASH Annual Meeting

**DOI:** 10.1186/s40164-023-00384-4

**Published:** 2023-03-11

**Authors:** Jieyu Xu, Hong-Hu Zhu

**Affiliations:** 1 Department of Hematology, College of Medicine, The First Affiliated Hospital, Zhejiang University, #79 Qingchun Road, Hangzhou, 310003 Zhejiang China; 2 Zhejiang Province Key Laboratory of Hematology Oncology Diagnosis and Treatment, Hangzhou, China; 3 Department of Hematology, Beijing Chao-Yang Hospital, Capital Medical University, No. 8 Gongtinanlu, Chaoyang District, Beijing, 100020 China

**Keywords:** T-cell acute lymphoblastic leukemia, Nelarabine, CAR T, Venetoclax

## Abstract

T-cell acute lymphoblastic leukemia (T-ALL) occurs in approximately 25–30% of adult ALL. Currently, treatment approaches for adult patients with T-ALL remain quite limited, with intensive multiagent chemotherapy serving as the backbone; however, the cure rate remains unsatisfactory. Thus, the discovery of novel therapeutic strategies, especially targeted therapies, is crucial. Clinical research efforts are now focused on adding targeted therapy that has selective activity for T-ALL to the backbone chemotherapy regimen. To date, nelarabine remains the only targeted agent specifically approved for relapsed T-ALL, and the use of nelarabine in the first-line regimen is still being studied. Meanwhile, a number of novel targeted therapies with low toxicity, such as immunotherapies, are being actively investigated. Chimeric antigen receptor (CAR) T-cell therapy for the treatment of T-cell malignancies has not been as successful as in treating B-ALL due to fratricide. Numerous approaches are now being designed to address this challenge. Novel therapies targeting molecular aberrations in T-ALL are also actively investigated. T-ALL lymphoblasts overexpress BCL2 protein, which makes it an intriguing therapeutic target. This review summarizes the latest updates on targeted treatment of T-ALL from the 2022 ASH annual meeting.

## To the editor,

Currently, treatment options for adult patients with T-cell acute lymphoblastic leukemia (T-ALL) are limited, mainly consisting of conventional intensive chemotherapy and hematopoietic stem cell transplantation [1]. Nelarabine, the only targeted agent approved for relapsed T-ALL, is now studied as a component of treatment. Immune-mediated therapies such as chimeric antigen receptor (CAR) T-cell therapy and small-molecule targeted agents such as BCL2 inhibitors are also being actively developed to improve outcomes [2, 3]. This review discusses the latest advances in targeted therapy for T-ALL and summarizes the highlights from the 2022 ASH annual meeting.

## Nelarabine-based treatment

The GMALL trial enrolled 281 newly diagnosed adult T-cell acute lymphoblastic leukemia/lymphoma (208 T-ALL; 73 T-LBL) patients to evaluate overall results with nelarabine (Abstract 51) [4]. With a 28-month median follow-up of this cohort, the 3-year overall survival (OS) for all patients was 78%. Seventy-eight high-risk patients received allogeneic stem cell transplantation, and the 3-year OS was 68%.

An intergroup phase II study was conducted to assess the pediatric regimen including nelarabine for newly diagnosed T-ALL in adolescents and young adults (AYAs) (Abstract 54) [5]. Sixty-two patients aged 15–24 years were enrolled. Fifty-six patients (90.3%) achieved complete remission (CR)/CRi. The 3-year event-free survival (EFS) and OS were 88.6% and 93.4%, respectively.

A retrospective study was designed to compare the outcomes of the nelarabine combination and monotherapy for relapsed or refractory (R/R) T-ALL/LBL (Abstract 4053) [6]. Forty-four patients who were ever treated with nelarabine for R/R T-ALL/LBL were enrolled. The CR rates were 62% and 40% in the combination and monotherapy groups (p = 0.21), respectively; the 2-year relapse-free survival (RFS) was 68.8% vs. 26.7%, and the 2-year OS was 52.9% vs. 8%, respectively.

## CAR‑T-based treatment

Base editing and protein expression blockers (PEBLs) facilitate knocking out or knocking down pan-T-cell surface biomarkers, such as CD3 and CD7, to avoid fratricide. A phase I/II study of CD7-targeted CAR-T-cell therapy enrolled 53 patients with R/R T-ALL/LBL who received naturally selected 7CAR-T-cell (NS7CAR) infusion (Abstract 980) [7]. NS7CARs were derived from bulk T cells without additional genetic manipulations. The 18-month OS and EFS were 75.0% and 53.1%, respectively. A total of 47/53 (88.7%) patients had mild (≤ grade II) cytokine release syndrome (CRS). Five patients experienced grade III CRS, and 1 patient experienced grade IV CRS.

A phase I clinical trial was conducted to evaluate a novel genome-edited anti-CD7 CAR-T-cell product, RD13-01, in ten R/R T-ALL/LBL patients (Abstract 1995) [8]. Four patients achieved CR, 4 died, and 2 showed no response. A total of 9/10 patients had grade I CRS, and 1/10 had grade 3 CRS.

Twenty R/R T-ALL/LBL patients were tested in a phase II trial of donor-derived CD7 CAR T cells (Abstract 2011) [9]. The authors designed a CD7-targeting CAR using the IntraBlock technology to prevent CD7 cell surface expression. The one-year progression-free survival (PFS) and OS rates were 62.3% and 60.0%, respectively. The percentages of patients experiencing grade 3 or higher CRS and grade 1–2 graft-versus-host disease (GvHD) were 10% and 40%, respectively.

Allogeneic T cells can be activated upon recognition of host tissue antigens by T-cell receptor/CD3 complex. To reduce the risk of causing GvHD, CD3 and CD7 PEBLs were developed simultaneously for intracellular protein retention, and this PCART7-CD3 PEBL showed minimal fratricide and a reduced risk of GvHD in a xenograft model (Abstract 975) [10]. Clinical data are needed to verify the feasibility of this treatment in humans.

## Venetoclax-based treatment

In a bench-to-clinic study, 3 R/R ETP-ALL patients and 4 newly diagnosed patients were administered venetoclax plus HAG (Homoharringtonine, low-dose cytarabine, G-CSF priming), which was named the V-HAG regimen (Abstract 942) [11]. The CR/CRi rate after the first cycle of treatment was 100%.

The V-HAG regimen was also evaluated in a phase II trial in patients with R/R ETP-ALL, similar to Abstract 942 (Abstract 1414) [12]. This study enrolled 7 R/R ETP-ALL patients. The CRc rate was 85.7% (6/7 patients).

Highlights in the treatment of T-ALL in the 2022 ASH annual meeting mainly focused on the use of targeted therapy to improve the first-line regimen. The selected studies, including all the most relevant and advanced studies on targeted therapy for T-ALL, are listed in Tables 1 and 2.

**Table 1:** Selected studies on targeted therapy combined with chemotherapy for T-ALL from 2022 ASH annual meeting

	Abstract #				
	51	54	4053	942	1414
Authors (references)	Goekbuget et al. [4]	Hatta et al. [5]	Shimony et al. [6]	Suo et al. [11]	Yu et al. [12]
Study agents	Nelarabine + cyclophosphamide	Nelarabine + L-ASP	Nelarabine monotherapy vs combination	Venetoclax + HAG	Venetoclax + HAG
Analysis	N/A	II	Retrospective	N/A	II
NCT No	NCT02881086	N/A	N/A	N/A	N/A
Study period	N/A	2011–2017	2006–2021	N/A	2021–2022
Age range, years	18–55	15–24	2–69	N/A	15–60
No. of patients	281 (208 T-ALL; 73 T-LBL)	62	44 (29 combination; 15 monotherapy)	7 (3 R/R ETP-ALL; 4 newly diagnosed)	7
MRD analysis used to assign risk/postremission therapy	Yes	Yes (< 10^–3^)	Yes	No	Yes (< 0.01%)
Outcome measure	1-year OS, 3-year OS, OS for SCT	3-year EFS, 3-year OS, 3-year CIR	CR, RFS, OS	CR/CRi rate	CR/CRi rate
Survival outcome	1-year OS: 89%; 3-year OS: 78%	3-year EFS: 88.6%; 3-year OS: 93.4%; 3-year CIR: 5.3%	CR: 55%; 2-year RFS: 60.5%; 2-year OS: 37.6%	CR/CRi rate: 100%	CR/CRi rate(after the first cycle): 85.7%; CR: 42.9%; CRi: 42.9%; PR: 14.3%; CR/CRi rate(after the second cycle): 100%
Summary	Early T-ALL subgroup appeared poorer outcomes	Risk stratification system in pediatric regimen was also effective for AYA patients	Nelarabine combination regimen led to better outcomes than monotherapy	V-HAG regimen in newly diagnosed and R/R ETP-ALL has led to favorable outcomes	VGHA regimen provides a new choice in the treatment of R/R ETP-ALL patients

MRD, minimal residual disease; OS, overall survival; EFS, event-free survival; CIR, cumulative incidence of relapse; RFS, relapse-free survival

**Table 2:** Selected studies on CAR-T-based treatment for T-ALL from 2022 ASH annual meeting

	Abstract #			
	980	1995	2011	975
Authors (references)	Zhang et al. [7]	Zhang et al. [8]	Tan et al. [9]	Wong et al. [10]
Study agents	NS7CAR-T	CD7 UCAR-T (RD13-01)	CD7 CAR-T	PCART7-CD3 PEBL
Analysis	I/II	I	II	N/A
NCT No	NCT04572308, NCT04916860	NCT04620655	NCT04689659	N/A
Study period	2020–2022	2020–2022	N/A	N/A
Age range, years	2–47	2–27	2–43	N/A
No. of patients	53 (34 T-ALL; 18 T/LBL)	10 (7 T-ALL; 3 T/LBL)	20	N/A
MRD analysis used to assign risk/postremission therapy	Yes	Yes	No	N/A
Outcome measure	OS, EFS	CR	ORR, 1-year PFS, 1-year OS	N/A
Survival outcome	18-month OS: 75.0%; 18-month EFS: 53.1%	CR: 80%	ORR: 90% at 3 months post-infusion; 1-year PFS: 62.3%; 1-year OS: 60.0%	N/A
Summary	NS7CAR therapy is safe and effective in R/R T-ALL/LBL patients with heavy pretreatment	RD13-01 product was safe and dose-dependently effective	Phase 2 trial of donor-derived CD7 CAR T cell therapy showed similar encouraging activity in treating R/R T-ALL with phase I trial	PCART7-CD3 PEBL showed minimized fratricide and a reduced risk of GvHD in a xenograft model. Clinical trials are needed to verify the feasibility in humans

ORR, objective response rate; PFS, progression-free survival

## Data Availability

Materials supporting the conclusions of this review have been included in the article.

## Abbreviations

T-ALL: T-cell acute lymphoblastic leukemia
CAR: Chimeric antigen receptor
OS: Overall survival
AYA: Adolescents and young adults
CR: Complete remission
EFS: Event-free survival
R/R: Relapsed or refractory
RFS: Relapse-free survival
PEBL: Protein expression blocker
NS7CAR: Naturally selected 7CAR-T-cell
CRS: Cytokine release syndrome
PFS: Progression-free survival
GvHD: Graft-versus-host disease
V-HAG: Venetoclax plus HAG
MRD: Minimal residual disease
CIR: Cumulative incidence of relapse
ORR: Objective response rate
